# R. Brooke Gwathmey

**Published:** 1884-12

**Authors:** 


### OBITUARY.

R. BROOKE GWATHMEY, of Darkville, Darlington Co., S. C., a student in the Dental Department of the University of Maryland. died in November, 1884, of typhoid fever, fever, after an illness of several weeks. Mr. Gwathmey was born in Virginia and at an early age went with his mother, his father having lost his life during the late war near Petersburg, Va., to reside in South Carolina. He had attended the entire session of 1883-1884, and during the period between sessions had been engaged in the practice of dentistry in Manchester, Va. Early in October last, Mr. Gwathmey returned to Baltimore and matriculated for his second course, at this time suffering from the effects of what was thought to be malaria, but which soon developed into the symptoms of typhoid fever. Mr. Gwathmey was a very promising student, and there was every reason to believe that had his life been spared he would have made an excellent dentist. His classmates, among whom he was held in the highest esteem, at a special meeting called for the purpose, adopted the following resolutions:

### UNIVERSITY OF MARYLAND DENTAL DEPARTMENT, CLASS 1884–1885.

At a special Meeting held by the above named class Nov. 29th, 1884, the following preamble and resolutions were unanimously adopted.

"Whereas, in view of the loss sustained by the decease of our friend and fellow-student R. Brooks Gwathmey, and of the still heavier loss sustained by those who were nearest and dearest to him; therefore, be it

"Resolved, that we sincerely condole with the relatives and friends of the deceased on the dispensation with which it has pleased Divine Providence to afflict them, and commend them for consolation to Him who orders all things for the best.

"Resolved, that this heartfelt testimonial of our sympathy and sorrow be forwarded to the relatives of our former classmate, and the same be published in the "Darlington News" of South Carolina, the "American Journal of Dental Science," the "Dental Cosmos," and a copy be placed in the Dental Department-Building of the Maryland University.

BROOKS RUTLEDGE, S. C.,
RALPH C. PURNELL, Md.,
CLAUDE D. BROWN, Va.,
AUGUSTUS H. CHAFEE, S. C.,
WILFRED A. PLEASANTS, Va.
FERDINAND GROSHANS, Md,
*Committee.*

---

